# Potential of Chemically Synthesized Oligosaccharides To Define the Carbohydrate Moieties of the Fungal Cell Wall Responsible for the Human Immune Response, Using Aspergillus fumigatus Galactomannan as a Model

**DOI:** 10.1128/mSphere.00688-19

**Published:** 2020-01-08

**Authors:** Sarah Sze Wah Wong, Vadim B. Krylov, Dmitry A. Argunov, Alexander A. Karelin, Jean-Phillipe Bouchara, Thierry Fontaine, Jean-Paul Latgé, Nikolay E. Nifantiev

**Affiliations:** a Unité des Aspergillus, Institut Pasteur, Paris, France; b Laboratory of Glycoconjugate Chemistry, N. D. Zelinsky Institute of Organic Chemistry, Russian Academy of Sciences, Moscow, Russia; c Groupe d’Etude des Interactions Hôte-Pathogène (EA 3142), UNIV Brest, Angers, France; Carnegie Mellon University

**Keywords:** *Aspergillus fumigatus*, aspergillosis, antibodies, cytokines, chemokines, immunology, *Aspergillus*, galactomannan, glycoarray

## Abstract

Methodologies to identify epitopes or ligands of the fungal cell wall polysaccharides influencing the immune response of human pathogens have to date been imperfect. Using the galactomannan (GM) of Aspergillus fumigatus as a model, we have shown that synthetic oligosaccharides of distinct structures representing key fragments of cell wall polysaccharides are the most precise tools to study the serological and immunomodulatory properties of a fungal polysaccharide.

## OPINION/HYPOTHESIS

Fungi are the only eukaryotes protected by a polysaccharide shell with an ambivalent function among pathogens, having a protective role against environmental stress and a negative role in the induction of an antifungal immune response (1). The carbohydrate fragments of the cell wall polysaccharide responsible for the induction of the immune response have often been poorly defined due to a lack of efficient tools. Currently, mutants lacking one polysaccharide due to the deletion of genes regulating its biosynthesis or polysaccharides purified from the cell wall are used. The first approach is indirect and does not take into account the putative compensatory reactions resulting from the gene deletion. The second approach results from purification of the cell wall polysaccharides. However, if it is possible to identify the target oligosaccharides in the case of long homogenous polysaccharides [and has been very appropriate in the identification of dectin-1, a receptor recognizing specifically the β-(1→3)-glucan chains (2)], this approach is more difficult when the composition of the repeating units of the polysaccharide is complex. This is the case for the galactomannan of Aspergillus fumigatus, which is composed of tetraose repeats with mannose units with α-(1→2) and α-(1→6) linkages bound to short side chains comprising β-(1→5)- and β-(1→6)-linked galactofuranose units of various lengths (3–5). It is even more difficult in the case of long polysaccharides without repeating units, such as the galactosaminogalactan of this species (6). The isolation of absolutely pure water-insoluble polysaccharide from the cell wall and the solubilization of immunoreactive oligosaccharides are difficult without structural modifications resulting from the harsh chemical extraction procedure required to solubilize the cell wall oligosaccharides.

In this report, we present an analysis of the oligosaccharides responsible for the immune response of the human host against the galactomannan (GM) of A. fumigatus. Even though galactofuran has been recognized as a potent *Aspergillus* immunogen (1, 7), the GM fragments modulating the host immune response have not been fully characterized. This study presents a new approach based on the use of synthetic oligosaccharides which allows a precise and unbiased identification of the carbohydrates responsible for the immune response.

### Glycoarray of oligosaccharides encompassing the complete structure of the galactomannan of Aspergillus fumigatus.

**FIG 1:**
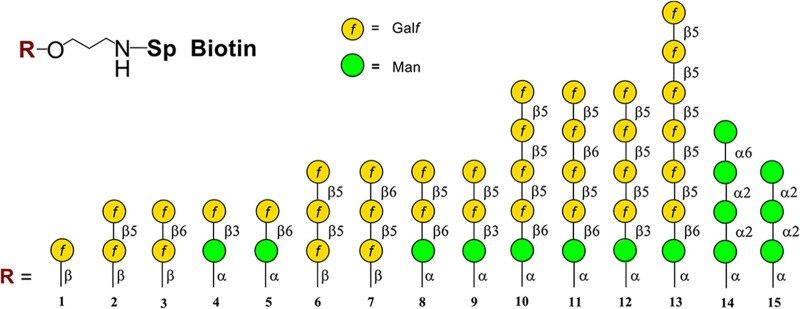
The structure of oligosaccharide ligands representing key structural elements of the galactofuranosylated side chains (ligands 1 to 13) and mannan backbone (ligands 14 and 15) of A. fumigatus galactomannan.

All fragments of the galactomannan molecule used in this study were chemically synthesized (Fig. 1). Oligosaccharides 1 to 13 related to galactofuranosylated side chains of galactomannan were obtained as previously described (4, 8, 9). Manno-oligosaccharides 14 and 15 (for preparation, see Text S1 in the supplemental material) represented a repeating unit of the mannan backbone of galactomannan. All oligosaccharides were biotinylated (10) and adsorbed to a streptavidin-coated plate to quantify the immune response.

10.1128/mSphere.00688-19.5 TEXT S1 Chemical synthesis of biotinylated mannotrioside and mannotetraoside representative of the mannan backbone of the galactomannan of A. fumigatus. Download Text S1, DOCX file, 0.1 MB. Copyright © 2020 Wong et al. 2020 Wong et al. This content is distributed under the terms of the Creative Commons Attribution 4.0 International license.

### The glycoarray with Gal*f*(1→5)Gal*f* blocks can be used to trace specific antibodies in sera from ABPA and CPA patients.

**FIG 2:**
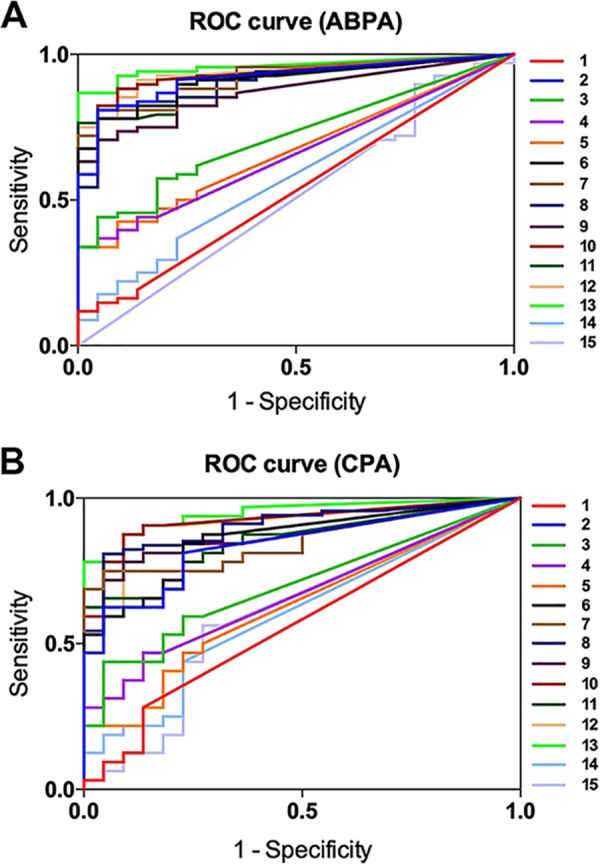
Results of enzyme-linked immunosorbent assay (ELISA) data with different oligosaccharide ligands related to A. fumigatus galactomannan and sera of aspergillosis patients. (A and B) The results are expressed as receiver operating characteristic (ROC) curves plotted for ABPA patient sera (A) and CPA patient sera (B) with regard to the control sera. Sensitivity represents the fraction of patient sera ranking as positive (true positive), and specificity represents the fraction of control sera ranking as negative (true negative). See Tables S1 and S2 for the statistical significance of the results.

No antibodies recognizing oligomannosides 14 and 15 were detected in the chronic pulmonary aspergillosis (CPA) or allergic bronchopulmonary aspergillosis (ABPA) patient sera (Fig. 2 and Text S2). Similarly, no antibodies recognizing ligands 1, 4, and 5 containing only one galactofuranose (Gal*f*) unit were detected in sera from controls and patients (Fig. 2). In contrast, the ligands with two Gal*f* units linked through a (1→5) linkage (ligands 2, 8, and 9), but not through a (1→6) linkage (ligand 3), gave antibody titers which were significantly higher in patients with ABPA or CPA than in the controls (*P* < 0.0001) (Fig. 2). Ligand 13 had the highest area under curve (AUC) value for both ABPA and CPA patient sera (Tables S1 to S4). However, the differences with oligosaccharides 6 to 13 were not statistically significant for patient discrimination (Tables S1 to S4). Interestingly, the presence of one to two Gal*f*(1→6)Gal*f* blocks in oligonucleotide-Gal*f* sequences with Gal*f*(1→5)Gal*f* blocks (ligands 7 and 11) did not affect their ability to distinguish between control and patient sera (Tables S1 to S4). The nature of the linkage between the oligonucleotide-Gal*f* chain and mannan (Man) unit [either a β-(1→3) linkage for ligands 9 and 12 or a β-(1→6) linkage for ligands 8 and 10] did not affect the level of antibody recognition (Fig. 2 and Tables S1 to S4).

10.1128/mSphere.00688-19.1 TABLE S1 Area under the curve (AUC) of the ROC curve and confidence interval (CI) obtained by plotting ELISA data generated with oligosaccharide fragments of the galactomannan of A. fumigatus and sera from ABPA patients. Download Table S1, DOCX file, 0.01 MB. Copyright © 2020 Wong et al. 2020 Wong et al. This content is distributed under the terms of the Creative Commons Attribution 4.0 International license.

10.1128/mSphere.00688-19.6 TEXT S2 Methods used to quantify the antibody titers in sera from ABPA and CPA patients against oligosaccharides representative of the galactomannan of A. fumigatus. Download Text S2, DOCX file, 0.01 MB. Copyright © 2020 Wong et al. 2020 Wong et al. This content is distributed under the terms of the Creative Commons Attribution 4.0 International license.

10.1128/mSphere.00688-19.2 TABLE S2 Statistical analysis comparing ROC curves obtained with sera from ABPA patients. Download Table S2, DOCX file, 0.01 MB. Copyright © 2020 Wong et al. 2020 Wong et al. This content is distributed under the terms of the Creative Commons Attribution 4.0 International license.

Early studies with immunocompetent patients with CPA or ABPA have shown that high antibody titers against GM were detected in the sera of these patients (7). Even though the immunodominance of the oligonucleotide-Gal*f* sequences in GM has been repeatedly shown in the past, the chemical nature of the epitope recognized in GM was not precisely identified (1). The use of a set of chemically synthesized oligosaccharides representing different parts of side oligonucleotide-Gal*f* sequences in GM has permitted the identification of the epitope recognized by the anti-A. fumigatus antibodies. Interestingly, no antibodies bound to the oligomannosides which are fragments of the repeating units of the mannan backbone of *Aspergillus* cell wall GM. This situation is entirely different from the mannan of Candida species. The *Candida* cell wall mannans are well-known antigens recognized in patient sera and have been used in the past for serotyping this species (11). The antibody response against *Candida* mannan is mainly associated with the linear α-(1→2)-linked side chains of the mannan core and the β-(1→2)-mannan oligosaccharides. Accordingly, the linkages between the mannose residues are essential for determining an immune response (11). Similarly, in A. fumigatus, the linkages between the galactofuranose residues and the size of the oligosaccharides are important since β-(1→6) linkages are not recognized unless they are intercalated with β-(1→5) linkages. These results showed that the recognition of the GM by the human antibodies is not dependent on a strict three-dimensional structure of the oligonucleotides. In addition, it is interesting to note that a Gal*f* disaccharide is a recognized antigenic epitope, while earlier studies have specified that the best oligosaccharide sequences usually recognized by antibodies have a degree of polymerization of 4 or 5.

### Oligosaccharides with Gal*f*(1→5)Gal*f* blocks can be used to understand the secretion of cytokines and chemokines by immune cells.

**FIG 3:**
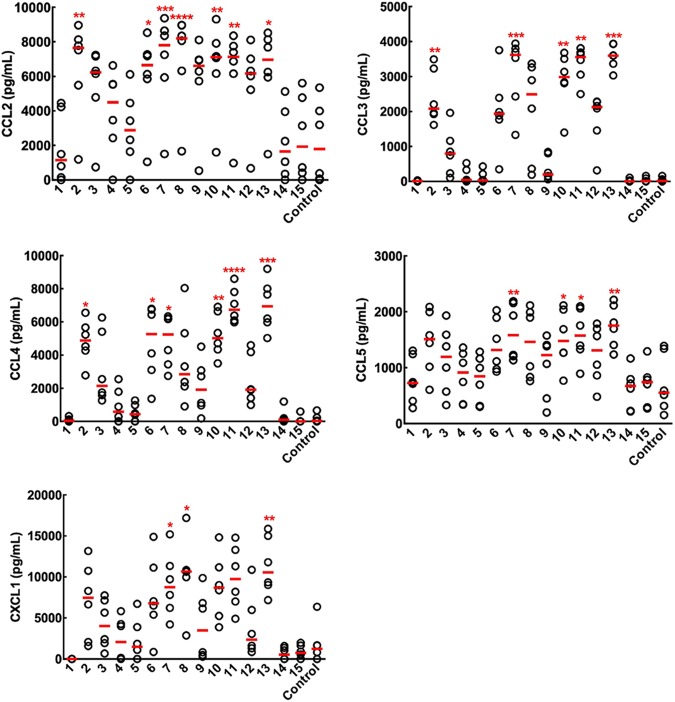
Chemokine induction by the oligosaccharides. The biotinylated oligosaccharides 1 to 15 were coated onto streptavidin microtiter plates, and PBMCs (six donors) were added to the microtiter plates in RPMI medium supplemented with 10% normal human serum. Controls are unstimulated PBMCs on streptavidin or nonstreptavidin microtiter plates. The chemokine concentrations obtained from each donor are plotted as dots, where the center red line indicates the median. The median chemokine concentrations were compared with those of the unstimulated PBMCs (control) by a Kruskal-Wallis test and Dunn’s multiple-comparison test (****, *P* < 0.0001; ***, *P* < 0.001; **, *P* < 0.01; *, *P* < 0.05).

**FIG 4:**
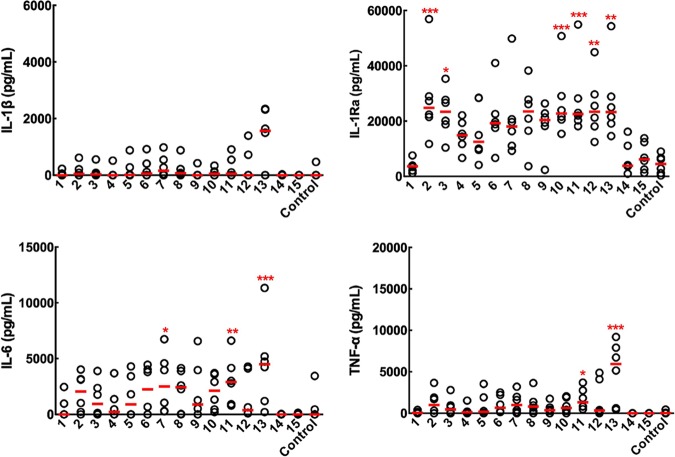
Cytokine induction by the oligosaccharides. The amounts of cytokines in the culture medium were examined by ELISA. The median chemokine concentrations were compared with those of the unstimulated PBMCs (control) by a Kruskal-Wallis test and Dunn’s multiple-comparison test (****, *P* < 0.0001; ***, *P* < 0.001; **, *P* < 0.01; *, *P* < 0.05).

The production of the specific cytokines interleukin 1 beta (IL-1β), IL-1Ra, IL-6, and tumor necrosis factor alpha (TNF-α) and chemokines CCL2, CCL3, CCL4, CCL5, and CXCL1 (known to be associated with aspergillosis [12, 13]) produced by peripheral blood mononuclear cells (PBMCs) in the presence of the oligosaccharides was quantified (Fig. 3 and 4 and Text S3). The findings and conclusions based on the analysis were basically similar for the chemokines and cytokines. Like for the antibody study, the ligands with only one Gal*f* unit (ligands 1 and 3 to 5) and oligomannosides (ligands 14 and 15) did not induce the production of cytokines and chemokines (Fig. 3). Similar amounts of cytokines and chemokines are produced in the presence of heat-inactivated serum or normal serum, indicating that the production of cytokines and chemokines is independent of the complement (data not shown). The analysis of the response of the trisaccharide and pentasaccharide 8 and 10, respectively, and their isomers, 9 and 12, respectively, showed that the nature of the chemical linkage of the galactofuran to the mannan chain significantly influenced the production of cytokines and chemokines (in contrast to the antibody data); the β-(1→3) linkage between tetra-Gal*f*- and Man was found to be detrimental to the production of cytokines. In contrast, the occurrence of a β-(1→6) linkage inside the galactofuran composed of (1→5) linkages did not influence cytokine and chemokine production (compare ligands 10 and 11). Like for the antibody epitope, galactofuranosides of two units with one β-(1→5) linkage between Gal*f* residues induced the production of cytokines and chemokines, although at a very limited amount, especially for the cytokines. Heptasaccharide 13 significantly induced the highest expression of all the chemokines and cytokines tested (Fig. 4).

10.1128/mSphere.00688-19.7 TEXT S3 Methods used to quantify the chemokine and cytokine response against oligosaccharides representative of the galactomannan of A. fumigatus. Download Text S3, DOCX file, 0.01 MB. Copyright © 2020 Wong et al. 2020 Wong et al. This content is distributed under the terms of the Creative Commons Attribution 4.0 International license.

The chemokines (CCL3, CCL2, and CCL5) followed in this study have been previously shown to be associated with aspergillosis since they are involved in the recruitment of neutrophils, platelets, and monocytes, but the molecules responsible for the chemokine production have not been identified (12, 13). GM has been previously recognized to induce the production of cytokines, but the conclusions on the pro- or anti-inflammatory function of GM have been controversial (14–16). The results indicated that in contrast to antibodies, the production of cytokines and chemokines was facilitated by longer chains of β-(1→5)-linked Gal*f* units. It will be now possible to analyze the presentation of the carbohydrate antigen on the major histocompatibility complexes. Similarly to patient antibodies, the production of cytokine and chemokines from PBMCs was not induced by GM-related oligomannosides. Again, this situation is opposite from the immune response against *Candida* spp. *Candida* mannan induces a strong immune cellular response. However, these studies were mostly based on the use of mannosyltransferase mutants rather than on the use of pure oligosaccharides, and conclusions were indirectly based on the lack of or modified response seen with the mutants rather than on a direct positive response of the cell toward pure oligosaccharides (17). Compensatory reactions resulting from the gene deletion often modify the composition of the cell wall; consequently, the immune response against the mutant may not result from the lack of the target polysaccharides but may be hidden by the modifications of the cell wall resulting from these compensatory reactions (1). Moreover, the discrepancy between immunological studies run with polysaccharides might be due to the insufficient purification of the polysaccharide used, since the precise characterization of the immunostimulatory activities of polysaccharides requires the use of pure polysaccharides (1, 18).

The purity of chemically synthesized oligosaccharides can be easily controlled and will make synthetic oligosaccharides a perfect tool to understand the immune role of carbohydrate moieties. In addition, the biotinylation of the oligosaccharides allows their quantitative immobilization onto streptavidin-coated devices, which can allow amplification of the response between the oligosaccharides and the immune cell surface. Even though many receptors (mannose-binding lectin, mannose receptor, dectin-2, dendritic cell-specific intercellular adhesion molecule-3 grabbing nonintegrin [DC-SIGN], intelectin-1, and even dectin-1) have been claimed to bind to GM (1, 14, 15, 19, 20), the binding has not been demonstrated biochemically. Such chemical tools will be the best use for such a demonstration.

### Conclusion.

Even though glycoarrays have been developed in the past (21, 22), they have not been focused specifically on fungal polysaccharides. Recent studies demonstrated that glycoarrays cannot be universal and that the carbohydrate molecules blotted on the array have to be specifically designed for the specific research project considered since not all oligosaccharides present in nature can be deposited on a single array. This study demonstrated that chemically synthesized oligosaccharides specifically designed to represent fragments of the fungal cell wall are appropriate tools to investigate precisely the immune response against this insoluble polysaccharide shell. We have in our hands oligosaccharides from all essential cell wall polysaccharides of A. fumigatus cell wall [α- and β-(1→3)-glucans, chitin, galactomannan, and galactosaminogalactan]. These oligosaccharides have now been bound to streptavidin beads to follow their internalization and their recognition at the phagosome level using a strategy previously developed by others to analyze the interactions between β-(1→3)-glucan and dectin-1 at the phagosomal level (23). Such approach will allow a precise definition of the mediators other than immunoglobulins, which can play a role in inducing an immune response against the cell wall components. In addition, such a strategy can also be applied to all cell wall polysaccharides from various human fungal pathogens.

10.1128/mSphere.00688-19.3 TABLE S3 Area under the curve (AUC) of the ROC curve and confidence interval (CI) obtained by plotting ELISA data generated with oligosaccharide fragments of the galactomannan of A. fumigatus and sera from CPA patients. Download Table S3, DOCX file, 0.01 MB. Copyright © 2020 Wong et al. 2020 Wong et al. This content is distributed under the terms of the Creative Commons Attribution 4.0 International license.

10.1128/mSphere.00688-19.4 TABLE S4 Statistical analysis comparing ROC curves obtained with the sera from CPA patients. Download Table S4, DOCX file, 0.01 MB. Copyright © 2020 Wong et al. 2020 Wong et al. This content is distributed under the terms of the Creative Commons Attribution 4.0 International license.

## References

[1] Latgé JP, Beauvais A, Chamilos G 2017 The cell wall of the human fungal pathogen *Aspergillus fumigatus*: biosynthesis, organization, immune response, and virulence. Annu Rev Microbiol 71:99–116. doi:10.1146/annurev-micro-030117-020406. 28701066

[2] Brown GD, Gordon S 2001 Immune recognition. A new receptor for beta-glucans. Nature 413:36–37. doi:10.1038/35092620. 11544516

[3] Latgé JP, Kobayashi H, Debeaupuis JP, Diaquin M, Sarfati J, Wieruszeski JM, Parra E, Bouchara JP, Fournet B 1994 Chemical and immunological characterization of the extracellular galactomannan of *Aspergillus fumigatus*. Infect Immun 62:5424–5433. 7960122 10.1128/iai.62.12.5424-5433.1994 PMC303284

[4] Krylov V, Argunov D, Solovev A, Petruk M, Gerbst A, Dmitrenok A, Shashkov A, Latgé J-P, Nifantiev N 2018 Synthesis of oligosaccharides related to galactomannans from *Aspergillus fumigatus* and their NMR spectral data. Org Biomol Chem 16:1188–1199. doi:10.1039/c7ob02734f. 29376539

[5] Kudoh A, Okawa Y, Shibata N 2015 Significant structural change in both O- and N-linked carbohydrate moieties of the antigenic galactomannan from *Aspergillus fumigatus* grown under different culture conditions. Glycobiology 25:74–87. doi:10.1093/glycob/cwu091. 25187160

[6] Fontaine T, Delangle A, Simenel C, Coddeville B, van Vliet SJ, van Kooyk Y, Bozza S, Moretti S, Schwarz F, Trichot C, Aebi M, Delepierre M, Elbim C, Romani L, Latgé J-P 2011 Galactosaminogalactan, a new immunosuppressive polysaccharide of *Aspergillus fumigatus*. PLoS Pathog 7:e1002372. doi:10.1371/journal.ppat.1002372. 22102815 PMC3213105

[7] Sarfati J, Monod M, Recco P, Sulahian A, Pinel C, Candolfi E, Fontaine T, Debeaupuis J-P, Tabouret M, Latgé J-P 2006 Recombinant antigens as diagnostic markers for aspergillosis. Diagn Microbiol Infect Dis 55:279–291. doi:10.1016/j.diagmicrobio.2006.02.002. 16626916

[8] Argunov DA, Krylov VB, Nifantiev NE 2015 Convergent synthesis of isomeric heterosaccharides related to the fragments of galactomannan from *Aspergillus fumigatus*. Org Biomol Chem 13:3255–3267. doi:10.1039/c4ob02634a. 25643073

[9] Argunov DA, Krylov VB, Nifantiev NE 2016 The use of pyranoside-into-furanoside rearrangement and controlled O(5)→O(6) benzoyl migration as the basis of a synthetic strategy to assemble (1→5)- and (1→6)-linked galactofuranosyl chains. Org Lett 18:5504–5507. doi:10.1021/acs.orglett.6b02735. 27759393

[10] Komarova BS, Wong SSW, Orekhova MV, Tsvetkov YE, Krylov VB, Beauvais A, Bouchara JP, Kearney JF, Aimanianda V, Latge JP, Nifantiev NE 2018 Chemical synthesis and application of biotinylated oligo-alpha-(1→3)-d-glucosides to study the antibody and cytokine response against the cell wall alpha-(1→3)-d-glucan of *Aspergillus fumigatus*. J Org Chem 83:12965–12976. doi:10.1021/acs.joc.8b01142. 30277398 PMC6461050

[11] Shibata N, Kobayashi H, Suzuki S 2012 Immunochemistry of pathogenic yeast, *Candida* species, focusing on mannan. Proc Jpn Acad Ser B Phys Biol Sci 88:250–265. doi:10.2183/pjab.88.250. PMC3410142 22728440

[12] Phadke AP, Mehrad B 2005 Cytokines in host defense against *Aspergillus*: recent advances. Med Mycol 43(Suppl 1):S173–S176. doi:10.1080/13693780500052099. 16110808

[13] Loeffler J, Ok M, Morton OC, Mezger M, Einsele H 2010 Genetic polymorphisms in the cytokine and chemokine system: their possible importance in allogeneic stem cell transplantation. Curr Top Microbiol Immunol 341:83–96. doi:10.1007/82_2010_22. 20397074

[14] Serrano-Gómez D, Domínguez-Soto A, Ancochea J, Jimenez-Heffernan JA, Leal JA, Corbí AL 2004 Dendritic cell-specific intercellular adhesion molecule 3-grabbing nonintegrin mediates binding and internalization of *Aspergillus fumigatus* conidia by dendritic cells and macrophages. J Immunol 173:5635–5643. doi:10.4049/jimmunol.173.9.5635. 15494514

[15] Toledano V, Hernández-Jiménez E, Cubillos-Zapata C, Flandez M, Álvarez E, Varela-Serrano A, Cantero R, Valles G, García-Rio F, López-Collazo E 2015 Galactomannan downregulates the inflammation responses in human macrophages via NFkappaB2/p100. Mediators Inflamm 2015:942517. doi:10.1155/2015/942517. 26441484 PMC4579314

[16] Chiodo F, Marradi M, Park J, Ram AF, Penades S, van Die I, Tefsen B 2014 Galactofuranose-coated gold nanoparticles elicit a pro-inflammatory response in human monocyte-derived dendritic cells and are recognized by DC-SIGN. ACS Chem Biol 9:383–389. doi:10.1021/cb4008265. 24304188

[17] Hall RA, Gow NA 2013 Mannosylation in *Candida albicans*: role in cell wall function and immune recognition. Mol Microbiol 90:1147–1161. doi:10.1111/mmi.12426. 24125554 PMC4112839

[18] Ferreira SS, Passos CP, Madureira P, Vilanova M, Coimbra MA 2015 Structure-function relationships of immunostimulatory polysaccharides: a review. Carbohydr Polym 132:378–396. doi:10.1016/j.carbpol.2015.05.079. 26256362

[19] Garred P, Genster N, Pilely K, Bayarri-Olmos R, Rosbjerg A, Ma YJ, Skjoedt MO 2016 A journey through the lectin pathway of complement-MBL and beyond. Immunol Rev 274:74–97. doi:10.1111/imr.12468. 27782323

[20] Wesener DA, Wangkanont K, McBride R, Song X, Kraft MB, Hodges HL, Zarling LC, Splain RA, Smith DF, Cummings RD, Paulson JC, Forest KT, Kiessling LL 2015 Recognition of microbial glycans by human intelectin-1. Nat Struct Mol Biol 22:603–610. doi:10.1038/nsmb.3053. 26148048 PMC4526365

[21] Geissner A, Reinhardt A, Rademacher C, Johannssen T, Monteiro J, Lepenies B, Thépaut M, Fieschi F, Mrázková J, Wimmerova M, Schuhmacher F, Götze S, Grünstein D, Guo X, Hahm HS, Kandasamy J, Leonori D, Martin CE, Sharavathi G, Parameswarappa S, Pasari S, Schlegel MK, Tanaka H, Guozhi X, Yang Y, Pereira CL, Anish C, Seeberger PH 2019 Microbe-focused glycan array screening platform. Proc Natl Acad Sci U S A 116:1958–1967. doi:10.1073/pnas.1800853116. 30670663 PMC6369816

[22] Rillahan CD, Paulson JC 2011 Glycan microarrays for decoding the glycome. Annu Rev Biochem 80:797–823. doi:10.1146/annurev-biochem-061809-152236. 21469953 PMC3116967

[23] Mansour MK, Tam JM, Khan NS, Seward M, Davids PJ, Puranam S, Sokolovska A, Sykes DB, Dagher Z, Becker C, Tanne A, Reedy JL, Stuart LM, Vyas JM 2013 Dectin-1 activation controls maturation of β-1,3-glucan-containing phagosomes. J Biol Chem 288:16043–16054. doi:10.1074/jbc.M113.473223. 23609446 PMC3668760

