# Supplementary material for: Potential of Chemically Synthesized Oligosaccharides To Define the Carbohydrate Moieties of the Fungal Cell Wall Responsible for the Human Immune Response, Using Aspergillus fumigatus Galactomannan as a Model
Source: mSphere. 2020 Jan 8;5(1):e00688-19. doi: 10.1128/mSphere.00688-19 (PMC6952192; doi:10.1128/mSphere.00688-19)
Supplement: TEXT S1 [file mSphere.00688-19-s0001.docx]

**Text S1.**

1. **Synthesis of biotinylated mannotetraoside 14**

*Reagents and conditions:* i: AgOTf, MS-4Å, CH_2_Cl_2_, -20 ˚C, 15 min, 96%; ii: NIS, TfOH, MS-4Å, CH_2_Cl_2_, -50˚C → -35˚C, 45 min, 68%; iii: 1) H_2_, Pd/C, EtOAc-MeOH, rt, 2h; 2) Amberlyst A-26 (OH^–^), H_2_O, rt, 16h, 74%; iv: C_6_F_5_-Spacer-Biotin, Et_3_N, rt, overnight, 53%.

**Ethyl 2,3,4,6-terta-O-benzoyl-α-D-mannopyranosyl-(1→6)-2,3,4-tri-O-benzoyl-1-thio-α-D-mannopyranoside (C).** Acceptor **B** (120 mg, 0.22 mmol) was dissolved in CH_2_Cl_2_ (2 mL) and stirred with 4Å-molecular sieves for 30 min. Then temperature was decreased to -25 ˚C and AgOTf (125 mg, 0.48 mmol) was added. After 5 min at -20 ˚C solution of donor **A** (165 mg, 0.25 mmol) in CH_2_Cl_2_ (2 mL) was slowly added dropwise. After 15 min at -15 ˚C the reaction was quenched by Et_3_N, filtered through *celite* layer, washed with Na_2_S_2_O_3_ (1 M aq.) and organic layer was concentrated *in vacuo*. Column chromatography (toluene:EtOAc = 15:1) afforded disaccharide **7** (235 mg, 96%) as a white amorphous solid. ^1^H NMR (600 MHz, CDCl_3_) δ 8.18 (d, *J* = 7.2 Hz, 2H, *o*-Ph*H*), 8.06 – 8.00 (m, 8H, *o*-Ph*H*), 7.87 (d, *J* = 7.5 Hz, 4H, *o*-Ph*H*), 7.60 – 7.14 (m, 21H, Ph*H*), 6.12 (t, *J* = 10.1 Hz, 1H, H‑4^II^), 6.10 (t, *J* = 10.1 Hz, 1H, H‑4^I^), 6.00 (dd, *J* = 10.1, 3.3 Hz, 1H, H‑3^II^), 5.91 (dd, *J* = 10.0, 3.4 Hz, 1H, H‑3^I^), 5.87 (dd, *J* = 3.5, 1.4 Hz, 1H, H‑2^I^), 5.79 (dd, *J* = 3.4, 1.8 Hz, 1H, H‑2^II^), 5.64 (br. s, 1H, H‑1^I^), 5.16 (d, *J* = 1.8 Hz, 1H, H‑1^II^), 4.84 (ddd, *J* = 10.3, 5.7, 1.9 Hz, 1H, H‑5^I^), 4.54 (dd, *J* = 12.1, 2.4 Hz, 1H, H‑6_a_^II^), 4.42 (ddd, *J* = 10.1, 4.3, 2.4 Hz, 1H, H‑5^II^), 4.34 (dd, *J* = 12.1, 4.3 Hz, 1H, H‑6_b_^II^), 4.19 (dd, *J* = 10.8, 5.8 Hz, 1H, H‑6_a_^I^), 3.80 (dd, *J* = 10.8, 2.0 Hz, 1H, H‑6_b_^I^), 2.94 – 2.81 (m, 2H, SC*H*_2_CH_3_), 1.48 (t, *J* = 7.4 Hz, 3H, SCH_2_C*H*_3_). ^13^C NMR (150 MHz, CDCl_3_) δ = 166.16 (Ph*C*(O)), 165.75 (Ph*C*(O)), 165.71 (Ph*C*(O)), 165.59 (2xPh*C*(O)), 165.39 (Ph*C*(O)), 165.33 (Ph*C*(O)), 133.66 (quat. Ph), 133.58 (quat. Ph), 133.54 (2x quat. Ph), 133.30 (quat. Ph), 133.21 (quat. Ph), 133.11 (quat. Ph), 130.14, 129.97, 129.94, 129.85, 129.83, 128.89, 128.69, 128.64, 128.55, 128.43 (Ph), 97.84 (C‑1^II^), 82.29 (C‑1^I^), 72.33 (C‑2^I^), 70.80 (C‑3^I^), 70.43 (C‑2^II^), 70.16 (C‑3^II^), 70.11 (C‑5^I^), 69.06 (C‑5^II^), 67.41 (C‑4^I^), 66.92 (C‑4^II^), 66.86 (C‑6^I^), 62.75 (C‑6^II^), 25.63 (S*C*H_2_CH_3_), 14.94 (SCH_2_*C*H_3_).

**3-Trifluoroacetamidopropyl 2,3,4,6-terta-O-benzoyl-α-D-mannopyranosyl-(1→6)-2,3,4-tri-O-benzoyl-α-D-mannopyranosyl-(1→2)-3,4,6-tri-O-benzyl-α-D-mannopyranosyl-(1→2)-3,4,6-tri-O-benzyl-α-D-mannopyranoside (E).** Carefully dried mixture of donor **C** (140 mg, 0.126 mmol) and acceptor **D** (Karelin et al, 2007) (87 mg, 0.084 mmol) was dissolved in CH_2_Cl_2_ (4 mL) and stirred with MS-4Å (200 mg ) for 25 min. Then temperature was decreased to -15 ˚C and NIS (57 mg, 0.25 mmol) was added. After 15 min temperature was decreased to -50 ˚C and TfOH (4 μL, 0.04 mmol) was added. The temperature was increased and kept around ‑35 ˚C for 45 min. The the reaction mixture was neutralized by 2 drops of pyridine, filtered through *celite* layer, washed with Na_2_S_2_O_3_ (1 M aq.) and organic layer was concentrated *in vacuo*. Column chromatography (petroleum ether:EtOAc = 2.2:1) afforded tetrasaccharide **9** (120 mg, 68%) as a white amorphous solid. ^1^H NMR (600 MHz, CDCl_3_) δ 8.27 – 8.21 (m, 2H, *o*-Ph*H*), 8.04 – 7.84 (m, 12H, *o*-Ph*H*), 7.64 – 7.13 (m, 49H, Ph*H*), 7.11 (t, *J* = 7.3 Hz, 1H, Ph*H*), 6.95 (t, *J* = 7.4 Hz, 1H, Ph*H*), 6.76 (t, *J* = 5.7 Hz, 1H, CH_2_N*H*TFA), 6.16 (t, *J* = 10.1 Hz, 1H, H‑4^III^), 6.10 (t, *J* = 10.0 Hz, 1H, H‑4^IV^), 6.02 (dd, *J* = 10.1, 3.2 Hz, 2H, H‑3^III^, H‑3^IV^), 5.94 (dd, *J* = 3.3, 1.9 Hz, 1H, H‑2^III^), 5.91 (dd, *J* = 3.2, 1.8 Hz, 1H, H‑2^IV^), 5.35 (d, *J* = 2.0 Hz, 1H, H‑1^II^), 5.14 (d, *J* = 1.8 Hz, 1H, H‑1^III^), 5.03 (d, *J* = 1.7 Hz, 1H, H‑1^IV^), 4.95 (d, *J* = 2.0 Hz, 1H, H‑1^I^), 4.94 (d, *J* = 10.5 Hz, 1H, PhC*H*_2_), 4.82 (d, *J* = 10.8 Hz, 1H, PhC*H*_2_), 4.77 (d, *J* = 11.3 Hz, 1H, PhC*H*_2_), 4.72 – 4.49 (m, 10H, 9xPhC*H*_2_, H‑5^III^), 4.35 (dd, *J* = 12.2, 2.3 Hz, 1H, H‑6_a_^IV^), 4.26 (ddd, *J* = 9.9, 3.8, 2.4 Hz, 1H, H‑5^IV^), 4.21 (dd, *J* = 12.1, 3.9 Hz, 1H, H‑6_b_^IV^), 4.16 (t, *J* = 2.4 Hz, 1H, H‑2^I^), 4.12 (t, *J* = 2.4 Hz, 1H, H‑2^II^), 4.05 (t, *J* = 9.4 Hz, 1H, H‑4^II^), 4.01 (dd, *J* = 9.2, 2.7 Hz, 1H, H‑3^II^), 3.99 – 3.95 (m, 1H, H‑5^II^), 3.91 (dd, *J* = 11.5, 4.0 Hz, 1H, H‑6_a_^III^), 3.88 (dd, *J* = 8.9, 3.0 Hz, 1H, H‑3^I^), 3.76 – 3.66 (m, 6H, H‑4^I^, H‑5^I^, H‑6_a_^I^, H‑6_a_^II^, H‑6_b_^I^, H‑6_b_^II^), 3.64 – 3.58 (m, 2H, H‑6_b_^III^, OC*H*_2_CH_2_CH_2_N), 3.35 – 3.26 (m, 2H, OC*H*_2_CH_2_CH_2_N, OCH_2_CH_2_C*H*_2_N), 3.19 – 3.12 (m, 1H, OCH_2_CH_2_C*H*_2_N), 1.69 – 1.55 (m, 2H, OCH_2_C*H*_2_CH_2_N). ^13^C NMR (150 MHz, CDCl_3_) δ = 166.11 (Ph*C*(O)), 165.66 (Ph*C*(O)), 165.62 (Ph*C*(O)), 165.56 (Ph*C*(O)), 165.52 (Ph*C*(O)), 165.29 (Ph*C*(O)), 165.25 (Ph*C*(O)), 138.57 (quat. *Ph*CH_2_), 138.46 (quat. *Ph*CH_2_), 138.39 (quat. *Ph*CH_2_), 138.35 (quat. *Ph*CH_2_), 138.31 (2x quat. *Ph*CH_2_), 133.51 (quat. Ph), 133.50 (quat. Ph), 133.46 (quat. Ph), 133.45 (quat. Ph), 133.16 (quat. Ph), 133.14 (quat. Ph), 133.0 (quat. Ph), 130.25, 130.05, 129.99, 129.93, 129.90, 129.87, 129.80, 129.70, 129.56, 129.28, 129.25, 128.96, 128.73, 128.54, 128.48, 128.46, 128.41, 128.37, 128.05, 128.03, 127.96, 127.80, 127.75, 127.71, 127.69 (Ph), 100.80 (C‑1^II^), 99.66 (C‑1^III^), 99.36 (C‑1^I^), 97.87 (C‑1^IV^), 80.07 (C‑3^I^), 79.29 (C‑3^II^), 77.44 (C‑2^II^), 75.60 (C‑2^I^), 75.42 (Ph*C*H_2_), 75.34 (Ph*C*H_2_), 75.21 (C‑4^I^), 74.77 (C‑4^II^), 73.55 (Ph*C*H_2_), 73.52 (Ph*C*H_2_), 72.68 (Ph*C*H_2_), 72.64 (Ph*C*H_2_), 72.43 (C‑5^II^), 72.33 (C‑5^I^), 70.62 (C‑3^III^, C‑3^IV^), 70.44 (C‑2^III^), 70.28 (C‑2^IV^), 69.64 (C‑6^I^), 69.57 (C‑6^II^), 69.47 (C‑5^III^), 69.00 (C‑5^IV^), 66.93 (C‑4^III^), 66.65 (C‑4^IV^), 66.09 (C‑6^III^), 65.87 (O*C*H_2_CH_2_CH_2_N), 62.63 (C‑6^IV^), 37.93(OCH_2_CH_2_*C*H_2_N), 28.23(OCH_2_*C*H_2_CH_2_N).

**3-Aminopropyl α-D-mannopyranosyl-(1→6)-α-D-mannopyranosyl-(1→2)-α-D-mannopyranosyl-(1→2)-α-D-mannopyranoside (F).** Palladium on carbon (125 mg) was added to a solution of tetrasaccharide **E** (120 mg, 0.0575 mmol) in EtOAc-MeOH 1:1 (3mL). Mixture was stirred for two hours in the atmosphere of hydrogen and then filtered. The catalyst was carefully washed with methanol and filtrate was consentrated. The residue was dissolved in water and treated with exchange resin Amberlyst A-26 (OH^–^) for 16 h. The resin was filtered off and the filtrate was concentrated. Deprotected tetrasaccharide **F** was isolated by gel chromatography on a TSK HW-40 (S) column in in 0.1 M aq. AcOH and lyophilized. Yield 30 mg (74%). [α]_D_^24^ = 66 (c 1, H_2_O). HRESIMS: found m/z 724.2870 [M+H]+; calcd for C_27_H_50_NO_21_ 724.2870. ^1^H NMR (600 MHz, H_2_O) δ 5.23 (s, 1H, H-1^II^), 5.08 (s, 1H, H-1^I^), 5.04 (s, 1H, H-1^III^), 4.93 (s, 1H, H-1^IV^), 4.11 (br s, 1H, H-2^II^), 4.07 (br s, 1H, H-2^III^), 3.99 (br s, 1H, H-2^IV^), 3.97-3.88 (m, 8H, H-2^I^, H-3^I^, H-3^II^, H-5^III^, H-6_a_^I^, H-6_a_^II^, H-6_a_^III^, H-6_a_^IV^), 3.88-3.83 (m, 3H, H-3^III^, H-3^IV^, OC*H*_2_CH_2_CH_2_N), 3.79-3.70 (m, 6H, H-4^III^, H-5^II^, H-6_b_^I^, H-6_b_^II^, H-6_b_^III^, H-6_b_^IV^), 3.70-3.64 (m, 4H, H-4^I^, H-4^II^, H-4^IV^, H-5^IV^), 3.64-3.57 (m, 2H, H-5^I^, OC*H*_2_CH_2_CH_2_N, 3.13 (m. 2H, OCH_2_CH_2_C*H*_2_N), 2.00 (m, 2H, OCH_2_C*H*_2_CH_2_N). ^13^C NMR (150 MHz, H_2_O) δ 103.6 (1C, C-1^III^), 102.2 (1C, C-1^II^), 100.8 (1C, C-1^IV^), 99.7 (1C, C-1^I^), 80.2 (2C, C-2^I^, C-2^II^), 75.0 (1C, C-5^II^), 74.3 (1C, C-5^I^), 74.2 (1C, C-5^IV^), 72.7 (1C, C-5^III^), 72.0, 71.9 (2C, C-3^III^, C-3^IV^), 71.7 (1C, C-3^I^), 71.5 (1C, C-2^IV^), 71.4 (2C, C-2^III^ C-3^II^), 68.6, 68.5, 68.3 (3C, C-4^I^, C-4^II^, C-4^IV^), 68.1 (1C, C-4^III^), 67.0 (1C, C-6^III^), 66.4 (1C, O*C*H_2_CH_2_CH_2_N), 62.7, (1C, C-6^IV^), 62.4, (2C, C-6^I^, C-6^II^), 39.0 (1C, OCH_2_CH_2_*C*H_2_N), 28.1 (1C, OCH_2_*C*H_2_CH_2_N).

**Synthesis of biotinylated mannotetroside 14.** A 0.062 M solution of active ester (C_6_F_5_-Spacer-Biotin) (Tsvetkov et al, 2011) in DMF (110 µL) and triethylamine (27 µL) were added to a solution of tetrasacharide **F** (3.8 mg, 5.3 µmol) in DMF (0.1 mL) and the mixture was stirred overnight at room temperature. Then the solvent was removed under reduced pressure and the residue was dissolved in water. The solution was passed through a nylon filter and the filtrate was purified by gel chromatography on a TSK HW-40(S) column (2×100 cm) in 0.05 M (NH_4_)_2_CO_3_, appropriate fractions were collected, and lyophilized to give **6** (3.1 mg, 53%) as a white amorphous powder. [α]_D_^24^ = 46.5 (c 0.2, H_2_O); HRMS (ESI) *m/z* found: 1285.5596 [M + H]^+^; calcd. for C_52_H_93_N_4_O_30_S: 1285.5590. ^1^H NMR (600 MHz, D_2_O; selected data): sugar moiety, 5.24 (s, 1H, H-1^II^), 5.07 (s, 1H, H-1^I^), 5.06 (s, 1H, H-1^III^), 4.94 (s, 1H, H-1^IV^), 4.12 (br s, 1H, H-2^II^), 4.08 (br s, 1H, H-2^III^), 4.01 (br s, 1H, H-2^IV^); biotin moiety, 4.62 (dd, 1H, *J*_6a,6trans_=5.1, *J*_6a,3a_ =7.8 Hz, H-6a), 4.35 (dd, 1H, *J*_3a,4_ =4.5, *J*_6a,3a_ =7.9 Hz, H-3a), 2.02 (dd, 1H, *J*_6a,6trans_=5.0, *J*_6trans,6cis_=13.1 Hz, H-6trans), 2.80 (d, 1H, *J*_6trans,6cis_=13.1 Hz, H-6cis).

1. **Synthesis of biotinylated mannotrioside 15**

*Reagents and conditions:* i: 1) Pd/C, H_2_¸ methanol, rt; 2) Amberlyst A-26 (OH^–^), H_2_O, rt, 84% for two step; ii: Et_3_N, DMF, rt, 46%.

**3-Aminopropyl α-D-mannopyranosyl-(1→2)-α-D-mannopyranosyl-(1→2)-α-D-mannopyranoside (H).** Palladium on carbon (375 mg) was added to a solution of trisaccharide **J** (305 mg, 0.208 mmol) in methanol (3mL). Mixture was stirred for two hours in the atmosphere of hydrogen and then filtered. The catalyst was carefully washed with methanol and filtrate was consentrated. The residue was dissolved in water and treated with exchange resin Amberlyst A-26 (OH^–^) for 16 h. The resin was filtered off and the filtrate was concentrated. Deprotected oligosaccharide **H** was purified by gel chromatography on a TSK HW-40 (S) column in in 0.1 M AcOH and lyophilized. Yield 120 mg (84%). [α]_D_^23^ = 55 (c 1, H_2_O). HRESIMS: found m/z 562.2331 [M+H]^+^; calcd for C_21_H_40_NO_16_ 562.2342. ^1^H NMR (600 MHz, H_2_O) δ 5.28 (s, 1H, H-1^II^), 5.09 (s, 1H, H-1^I^), 5.04 (s, 1H, H-1^III^), 4.11 (br s, 1H, H-2^II^), 4.07 (br s, 1H, H-2^III^), 3.97-3.93 (m, 2H, H-2^I^, H-3^II^), 3.93-3.87 (m, 4H, H-3^I^, H-6_a_^I^, H-6_a_^II^, H-6_a_^III^), 3.86-3.82 (m, 2H, H-3^III^, OC*H*_2_CH_2_CH_2_N), 3.79-3.72 (5H, H-5^II^, H-5^III^, H-6_b_^I^, H-6_b_^II^, H-6_b_^III^), 3.72-3.63 (m, 3H, H-4^I^, H-4^II^, H-4^III^), 3.62-3.58 (m, 2H, H-5^I^, OC*H*_2_CH_2_CH_2_N), 3.12 (m. 2H, OCH_2_CH_2_C*H*_2_N), 2.00 (m, 2H, OCH_2_C*H*_2_CH_2_N). ^13^C NMR (150 MHz, H_2_O) δ 103.7 (1C, C-1^III^), 102.1 (1C, C-1^II^), 99.6 (1C, C-1^I^), 80.4 (1C, C-2^I^), 80.0 (1C, C-2^II^), 74.8, 74.7 (2C, C-5^II^, C-5^III^), 74.3 (1C, C-5^I^), 71.8 (1C, C-3^III^), 71.6 (1C, C-3^I^), 71.4 (2C, C-2^III^, C-3^II^), 68.6, 68.4, 68.3 (3C, C-4^I^, C-4^II^, C-4^III^), 66.5 (1C, O*C*H_2_CH_2_CH_2_N), 62.7, 62.6, 62.4 (3C, C-6^I^, C-6^II^, C-6^III^), 38.9 (1C, OCH_2_CH_2_*C*H_2_N), 28.1 (1C, OCH_2_*C*H_2_CH_2_N).

**Synthesis of biolinylated mannotrioside 15.** A 0.062 M solution of active ester (C_6_F_5_-Spacer-Biotin) (Tsvetkov et al, 2011) in DMF (89 µL) and triethylamine (40 µL) were added to a solution of trisacharide **H** (4.7 mg, 8.3 µmol) in DMF (0.1 mL) and the mixture was stirred overnight at room temperature. Then the solvent was removed under reduced pressure and the residue was dissolved in water. The solution was passed through a nylon filter and the filtrate was purified by gel chromatography on a TSK HW-40(S) column (2×100 cm) in 0.05 M (NH_4_)_2_CO_3_, appropriate fractions were collected, and lyophilized to give 4 (3.6 mg, 46%) as a white amorphous powder. HRMS (ESI) *m/z* found: 1145.4845 [M + Na]^+^; calcd. for C_46_H_82_N_4_NaO_25_S: 1145.4881. ^1^H NMR (500 MHz, D_2_O; selected data): sugar moiety, 5.28 (s, 1H, H-1^II^), 5.07 (s, 1H, H-1^I^), 5.05 (s, 1H, H-1^III^), 4.11 (br s, 1H, H-2^II^), 4.06 (br s, 1H, H-2^III^), biotin moiety, 4.61 (dd, 1H, *J*_6a,6trans_=5.1, *J*_6a,3a_ =7.6 Hz, H-6a), 4.42 (dd, 1H, *J*_3a,4_ =4.5, *J*_6a,3a_ =7.7 Hz, H-3a), 3.00 (dd, 1H, *J*_6a,6trans_=5.0, *J*_6trans,6cis_=13.0 Hz, H-6trans), 2.79 (d, 1H, *J*_6trans,6cis_=13.1 Hz, H-6cis).

**References**

1. Karelin AA, Tsvetkov YE, Kogan G, Bystricky S, Nifantiev NE. Synthesis of oligosaccharide fragments of mannan from *Candida albicans* cell wall and their BSA conjugates. Russ J Bioorganic Chem. 2007;33: 110–121.

2. Tsvetkov YE, Burg-Roderfeld M, Loers G, Ardá A, Sukhova EV, Khatuntseva EA, et al. Synthesis and molecular recognition studies of the HNK-1 trisaccharide and related oligosaccharides. The specificity of monoclonal anti-HNK-1 antibodies as assessed by surface plasmon resonance and STD NMR. J Am Chem Soc. 2011;134: 426–435.
