# Supplementary material for: Potential of Chemically Synthesized Oligosaccharides To Define the Carbohydrate Moieties of the Fungal Cell Wall Responsible for the Human Immune Response, Using Aspergillus fumigatus Galactomannan as a Model
Source: mSphere. 2020 Jan 8;5(1):e00688-19. doi: 10.1128/mSphere.00688-19 (PMC6952192; doi:10.1128/mSphere.00688-19)
Supplement: TABLE S1 [file mSphere.00688-19-st001.docx]

**Table S1**

| Oligo | AUC (95% CI) | p-value |
| --- | --- | --- |
| 1 | 0.534 (0.400 - 0.668) | 0.636 |
| 2 | 0.917 (0.859 - 0.976) | **** |
| 3 | 0.718 (0.611 - 0.825) | ** |
| 4 | 0.660 (0.545 - 0.775) | * |
| 5 | 0.670 (0.556 - 0.784) | * |
| 6 | 0.910 (0.851 - 0.970) | **** |
| 7 | 0.920 (0.864 - 0.975) | **** |
| 8 | 0.905 (0.842 - 0.969) | **** |
| 9 | 0.870 (0.799 - 0.942) | **** |
| 10 | 0.929 (0.875 - 0.983) | **** |
| 11 | 0.912 (0.854 - 0.970) | **** |
| 12 | 0.936 (0.887 - 0.985) | **** |
| 13 | 0.959 (0.920 - 0.997) | **** |
| 14 | 0.575 (0.444 - 0.707) | 0.291 |
| 15 | 0.514 (0.371 - 0.657) | 0.847 |

The area under curve (AUC) and the 95% confidence interval (CI) of the ROC curves of each oligosaccharide are shown here. The p-values are shown (* p < 0.05, ** p < 0.01, *** p < 0.001 , **** p < 0.0001).
