# Supplementary material for: Potential of Chemically Synthesized Oligosaccharides To Define the Carbohydrate Moieties of the Fungal Cell Wall Responsible for the Human Immune Response, Using Aspergillus fumigatus Galactomannan as a Model
Source: mSphere. 2020 Jan 8;5(1):e00688-19. doi: 10.1128/mSphere.00688-19 (PMC6952192; doi:10.1128/mSphere.00688-19)
Supplement: TEXT S2 [file mSphere.00688-19-s0002.docx]

## **Text S2.**

A total of 122 serum samples were collected with written consent from healthy individuals and aspergillosis patients from Angers Hospital (Angers, France). Twenty-two serum samples were collected from 21 healthy individuals as the control group. A total of 117 serum samples were collected from 56 aspergillosis patients, which include allergic bronchopulmonary aspergillosis (ABPA; 35 patients, 68 serum samples) and chronic pulmonary aspergillosis (CPA; 14 patients, 32 serum samples).

After biotinylation, the absence of endotoxin in the oligosaccharides was verified using the LAL assay (ThermoScientific). The biotinylated oligosaccharides were added to streptavidin-coated plates (ThermoScientific) at the concentration of 100 pM in PBS supplemented with 0.1% bovine serum albumin (Sigma-Aldrich) and 0.05% Tween 20 (PBS-BSA-Tween) and incubated for 2h. The plates were then washed three times with 200 µL/well wash buffer (PBS supplemented with 0.05% Tween 20). The sera of aspergillosis patients were diluted 1:500 in PBS-BSA-Tween and added to the plates coated with the biotinylated oligosaccharides. After incubation at room temperature for 1h, the plates were washed three times with 200 µL/well wash buffer. Anti-human IgG antibody (Fc specific) conjugated to peroxidase (Sigma-Aldrich) was added to the plates in dilution 1:1000 in PBS-BSA-Tween and incubated for 45 min. After washing, *O*-phenylenediamine-dihydrochloride (OPD) was added and the colour development was stopped by 4% sulphuric acid. The optical density at 492 nm was measured. To evaluate the predictive power of the ligands in measuring antibodies in patient sera, the receiver operating characteristic (ROC) curves of each ligand were constructed against their corresponding control sera. The values of area under the curve (AUC) with 95% confidence interval (CI) were also shown. The significance of differences between the AUC of each ligand was tested according to the method by Hanley and McNeil (1982). A p-value less than 0.05 is considered significant.

**Reference**

Hanley JA, McNeil BJ. 1982. The meaning and use of the area under a receiver operating characteristic (ROC) curve. Radiology 143:29-36.
