# Supplementary material for: Potential of Chemically Synthesized Oligosaccharides To Define the Carbohydrate Moieties of the Fungal Cell Wall Responsible for the Human Immune Response, Using Aspergillus fumigatus Galactomannan as a Model
Source: mSphere. 2020 Jan 8;5(1):e00688-19. doi: 10.1128/mSphere.00688-19 (PMC6952192; doi:10.1128/mSphere.00688-19)
Supplement: TABLE S2 [file mSphere.00688-19-st002.docx]

**Table S2**.

|  | 1 | 2 | 3 | 4 | 5 | 6 | 7 | 8 | 9 | 10 | 11 | 12 | 13 | 14 | 15 |
| --- | --- | --- | --- | --- | --- | --- | --- | --- | --- | --- | --- | --- | --- | --- | --- |
| 1 |  | **** | * | 0.162 | 0.130 | **** | **** | **** | **** | **** | **** | **** | **** | 0.666 | 0.841 |
| 2 |  |  | ** | **** | *** | 0.875 | 0.953 | 0.790 | 0.321 | 0.768 | 0.906 | 0.625 | 0.247 | **** | **** |
| 3 |  |  |  | 0.469 | 0.547 | ** | ** | ** | * | *** | ** | *** | **** | 0.100 | * |
| 4 |  |  |  |  | 0.904 | *** | **** | *** | ** | **** | *** | **** | **** | 0.342 | 0.118 |
| 5 |  |  |  |  |  | *** | *** | *** | ** | **** | *** | **** | **** | 0.287 | 0.094 |
| 6 |  |  |  |  |  |  | 0.825 | 0.910 | 0.396 | 0.647 | 0.968 | 0.511 | 0.181 | **** | **** |
| 7 |  |  |  |  |  |  |  | 0.742 | 0.285 | 0.807 | 0.856 | 0.658 | 0.255 | **** | **** |
| 8 |  |  |  |  |  |  |  |  | 0.471 | 0.576 | 0.879 | 0.451 | 0.160 | **** | **** |
| 9 |  |  |  |  |  |  |  |  |  | 0.198 | 0.374 | 0.137 | * | *** | **** |
| 10 |  |  |  |  |  |  |  |  |  |  | 0.674 | 0.849 | 0.384 | **** | **** |
| 11 |  |  |  |  |  |  |  |  |  |  |  | 0.535 | 0.192 | **** | **** |
| 12 |  |  |  |  |  |  |  |  |  |  |  |  | 0.483 | **** | **** |
| 13 |  |  |  |  |  |  |  |  |  |  |  |  |  | **** | **** |
| 14 |  |  |  |  |  |  |  |  |  |  |  |  |  |  | 0.535 |
| 15 |  |  |  |  |  |  |  |  |  |  |  |  |  |  |  |

The comparisons between AUC obtained from the ABPA sera were performed using the method by Hanley and McNeil. The p-values are shown (* p < 0.05, ** p < 0.01, *** p < 0.001, **** p < 0.0001).
