# Supplementary material for: Potential of Chemically Synthesized Oligosaccharides To Define the Carbohydrate Moieties of the Fungal Cell Wall Responsible for the Human Immune Response, Using Aspergillus fumigatus Galactomannan as a Model
Source: mSphere. 2020 Jan 8;5(1):e00688-19. doi: 10.1128/mSphere.00688-19 (PMC6952192; doi:10.1128/mSphere.00688-19)
Supplement: TEXT S3 [file mSphere.00688-19-s0003.docx]

## **Text S3**

Whole blood samples of six healthy donors were obtained with written consent in Hôpital Saint-Louis through Etablissement Français du Sang (Paris, France). The use of this material was approved by the ethics committees of Institut Pasteur and the Etablissement Français du Sang (convention 12/EFS/023). Human peripheral blood mononuclear cells (PBMCs) were isolated from the whole blood samples by Ficoll gradient. The biotinylated oligosaccharides were coated onto streptavidin microtiter plates of 96 wells as described in text S2. Each well was supplied with 2 x 10^6^ PBMCs in RPMI medium supplemented with 10% normal human serum. Preliminary assays have shown that similar amounts of cytokines and chemokines are produced in presence of heat-inactivated serum or normal serum indicating that the production of cytokines and chemokines is independent of the complement. After incubation of 24h at 37 ^o^C, the supernatant was collected and stored at -20^o^C for further ELISA analysis. The cytokines IL-1β, IL-1Ra, IL-6 and TNF-α and the chemokines CCL2, 3, 4, 5 and CXCL1) produced by PBMCs were quantified by R&D system ELISA kits. Statistical analyses were performed using the GraphPad Prism software.
