# Supplementary material for: Potential of Chemically Synthesized Oligosaccharides To Define the Carbohydrate Moieties of the Fungal Cell Wall Responsible for the Human Immune Response, Using Aspergillus fumigatus Galactomannan as a Model
Source: mSphere. 2020 Jan 8;5(1):e00688-19. doi: 10.1128/mSphere.00688-19 (PMC6952192; doi:10.1128/mSphere.00688-19)
Supplement: TABLE S3 [file mSphere.00688-19-st003.docx]

**Table S3.**

| Oligo | AUC (95% CI) | p-value |
| --- | --- | --- |
| 1 | 0.565 (0.409 - 0.720) | 0.423 |
| 2 | 0.839 (0.732 - 0.944) | **** |
| 3 | 0.700 (0.562 - 0.838) | * |
| 4 | 0.666 (0.524 - 0.809) | * |
| 5 | 0.628 (0.479 - 0.777) | 0.113 |
| 6 | 0.859 (0.761 - 0.958) | **** |
| 7 | 0.851 (0.748 - 0.954) | **** |
| 8 | 0.865 (0.772 - 0.959) | **** |
| 9 | 0.868 (0.770 - 0.966) | **** |
| 10 | 0.923 (0.849 - 0.998) | **** |
| 11 | 0.851 (0.750 - 0.952) | **** |
| 12 | 0.916 (0.838 - 0.994) | **** |
| 13 | 0.947 (0.891 - 1.004) | **** |
| 14 | 0.601 (0.448 - 0.754) | 0.211 |
| 15 | 0.602 (0.444 - 0.761) | 0.205 |

The area under curve (AUC) and the 95% confidence interval (CI) of the ROC curves of each oligosaccharide are shown here. The p-values are shown (* p < 0.05, ** p < 0.01, *** p < 0.001 , **** p < 0.0001).
