# Supplementary material for: Potential of Chemically Synthesized Oligosaccharides To Define the Carbohydrate Moieties of the Fungal Cell Wall Responsible for the Human Immune Response, Using Aspergillus fumigatus Galactomannan as a Model
Source: mSphere. 2020 Jan 8;5(1):e00688-19. doi: 10.1128/mSphere.00688-19 (PMC6952192; doi:10.1128/mSphere.00688-19)
Supplement: TABLE S4 [file mSphere.00688-19-st004.docx]

**Table S4.**

|  | 1 | 2 | 3 | 4 | 5 | 6 | 7 | 8 | 9 | 10 | 11 | 12 | 13 | 14 | 15 |
| --- | --- | --- | --- | --- | --- | --- | --- | --- | --- | --- | --- | --- | --- | --- | --- |
| 1 |  | ** | 0.201 | 0.345 | 0.565 | ** | ** | ** | ** | **** | ** | **** | **** | 0.744 | 0.739 |
| 2 |  |  | 0.120 | 0.058 | * | 0.772 | 0.865 | 0.707 | 0.685 | 0.196 | 0.864 | 0.248 | 0.074 | * | * |
| 3 |  |  |  | 0.736 | 0.484 | 0.066 | 0.087 | 0.053 | 0.052 | ** | 0.084 | ** | ** | 0.344 | 0.361 |
| 4 |  |  |  |  | 0.715 | * | * | * | * | ** | * | ** | **** | 0.540 | 0.557 |
| 5 |  |  |  |  |  | * | * | ** | ** | *** | * | *** | **** | 0.805 | 0.818 |
| 6 |  |  |  |  |  |  | 0.907 | 0.934 | 0.904 | 0.309 | 0.906 | 0.381 | 0.128 | ** | ** |
| 7 |  |  |  |  |  |  |  | 0.841 | 0.815 | 0.264 | 1.000 | 0.327 | 0.108 | ** | ** |
| 8 |  |  |  |  |  |  |  |  | 0.968 | 0.339 | 0.840 | 0.417 | 0.140 | ** | ** |
| 9 |  |  |  |  |  |  |  |  |  | 0.377 | 0.813 | 0.456 | 0.169 | ** | ** |
| 10 |  |  |  |  |  |  |  |  |  |  | 0.257 | 0.887 | 0.613 | *** | *** |
| 11 |  |  |  |  |  |  |  |  |  |  |  | 0.321 | 0.102 | ** | ** |
| 12 |  |  |  |  |  |  |  |  |  |  |  |  | 0.516 | *** | *** |
| 13 |  |  |  |  |  |  |  |  |  |  |  |  |  | **** | **** |
| 14 |  |  |  |  |  |  |  |  |  |  |  |  |  |  | 0.990 |
| 15 |  |  |  |  |  |  |  |  |  |  |  |  |  |  |  |

The comparisons between AUC obtained from the CPA sera were performed using the method by Hanley and McNeil. The p-values are shown (* p < 0.05, ** p < 0.01, *** p < 0.001 , **** p < 0.0001).
